# School closures reduced social mixing of children during COVID-19 with implications for transmission risk and school reopening policies

**DOI:** 10.1098/rsif.2020.0970

**Published:** 2021-04-14

**Authors:** Jennifer R. Head, Kristin L. Andrejko, Qu Cheng, Philip A. Collender, Sophie Phillips, Anna Boser, Alexandra K. Heaney, Christopher M. Hoover, Sean L. Wu, Graham R. Northrup, Karen Click, Naomi S. Bardach, Joseph A. Lewnard, Justin V. Remais

**Affiliations:** ^1^ Division of Epidemiology, School of Public Health, University of California, Berkeley, CA, USA; ^2^ Division of Environmental Health Sciences, School of Public Health, University of California, Berkeley, CA, USA; ^3^ College of Letters and Science, University of California, Berkeley, CA, USA; ^4^ Center for Computational Biology, College of Engineering, University of California, Berkeley, CA, USA; ^5^ Division of Infectious Diseases and Vaccinology, School of Public Health, University of California, Berkeley, CA, USA; ^6^ Department of Pediatrics, School of Medicine, University of California, San Francisco, CA, USA

**Keywords:** children social networks, school closures and reopening, COVID-19, SARS-CoV-2, contact rate, transmission model

## Abstract

School closures may reduce the size of social networks among children, potentially limiting infectious disease transmission. To estimate the impact of K–12 closures and reopening policies on children's social interactions and COVID-19 incidence in California's Bay Area, we collected data on children's social contacts and assessed implications for transmission using an individual-based model. Elementary and Hispanic children had more contacts during closures than high school and non-Hispanic children, respectively. We estimated that spring 2020 closures of elementary schools averted 2167 cases in the Bay Area (95% CI: −985, 5572), fewer than middle (5884; 95% CI: 1478, 11.550), high school (8650; 95% CI: 3054, 15 940) and workplace (15 813; 95% CI: 9963, 22 617) closures. Under assumptions of moderate community transmission, we estimated that reopening for a four-month semester without any precautions will increase symptomatic illness among high school teachers (an additional 40.7% expected to experience symptomatic infection, 95% CI: 1.9, 61.1), middle school teachers (37.2%, 95% CI: 4.6, 58.1) and elementary school teachers (4.1%, 95% CI: −1.7, 12.0). However, we found that reopening policies for elementary schools that combine universal masking with classroom cohorts could result in few within-school transmissions, while high schools may require masking plus a staggered hybrid schedule. Stronger community interventions (e.g. remote work, social distancing) decreased the risk of within-school transmission across all measures studied, with the influence of community transmission minimized as the effectiveness of the within-school measures increased.

## 1. Introduction

In response to the coronavirus (COVID-19) pandemic, long-term K–12 school closures were implemented across many settings to reduce the risk of severe acute respiratory syndrome coronavirus 2 (SARS-CoV-2) transmission among students, teachers and family members. However, the long-term continuation of school closures poses a grave threat to healthy child development [1–3] and may exacerbate existing racial and socioeconomic gaps in school achievement [4] or nutrition [5]. The lack of data on children's social behaviour during long-term closures has prevented robust assessment of school closure policies. Contact surveys among children have found weakened contact networks during short-term school closures [6], weekends and holidays [7], but the impact of long-term COVID-19-related school closures on children's contact networks remains unclear. Much of our understanding about social contact patterns during the COVID-19 pandemic has been limited to adult behaviours [8–10] with only one study quantifying social contacts among children [11].

COVID-19 outbreaks within schools that held in-person instruction without physical distancing modifications [12] highlight the need to rigorously determine—and enact—effective risk reduction measures. A US modelling study estimates that reductions in within-school mixing of children via classroom cohorts or hybrid schedules may limit risk of school-attributable infection by four- to sevenfold, respectively [13]. Modification of individual behaviours, such as wearing face masks [13–15], quarantine of contacts of sick individuals [16] and increased testing [17], is also expected to reduce school-based transmission. K–12 schools in North Carolina reported only 32 school-acquired infections among over 90 000 students that attended in-person schooling with precautions involving universal masking, daily symptom monitoring and a 2-day-per-week hybrid schedule [17]. Nevertheless, these studies may be limited by non-detection of asymptomatic transmission. The REACT (REal-time Assessment of Community Transmission) study, in the UK, assessed time trend data of both asymptomatic and symptomatic infection, finding that children aged 13–17 years had a similar infection prevalence to working age adults, and only slightly higher than children aged 5–12 years [18]. Increases in prevalence were observed in children, and other age groups, after the reopening of schools in September 2020 [18]; however, national reopening guidelines recommended that masks should not be used in any classroom [19].

Differences in school size and social mixing patterns across age groups, as well as possible differences in susceptibility and transmissibility by age and the variants circulating [20], may contribute to heterogeneity in transmission risk across schooling levels. Meta-analysis found that children below 10 years of age had 48% lower odds of secondary infection of SARS-CoV-2 than adults, whereas there was no significant difference between adolescents and adults [21]. Secondary attack rates derived from contact tracing data of child index cases are conflicting, and it remains unclear whether children and adults are similarly infectious [22–26]. Empirically, differences in transmission between elementary (ages 5–10)- and high school-aged children (ages 14–18) are observed. In England, a study of over 9 million adults found that living with a child aged 12–18 years, but not a child 0–11 years, was associated with a slightly increased risk of SARS-CoV-2 infection [27]. Serological testing prior to closures in France revealed limited evidence of secondary transmission within primary schools [28], but a high seroprevalence of 38% among high school students and 43% among high school teachers after reopening [28]. Accordingly, it is imperative that the impact of school closures be evaluated separately for elementary, middle (ages 11–13) and high schools. At the same time, teachers and staff may experience a higher risk of infection than students. In the UK, monitoring of over 19 000 schools between 1 June and 17 July revealed 210 cases across 55 outbreaks [29]. Staff made up 73% of cases, and 26 outbreaks were driven by staff-to-staff transmission [29]. Therefore, it is also critical to assess impacts in different school community groups—teachers, students and family members.

The objectives of this study were to: (i) estimate social contact patterns among school-aged children during Bay Area (California) COVID-19-related school closures; (ii) estimate the cumulative incidence of COVID-19 throughout the 2020 spring semester under counterfactual scenarios had schools or workplaces remained open, or social distancing policies not been enacted; and (iii) estimate the effect of various school reopening strategies in Bay Area schools by grade level and across a new school semester. We focus our study on the Bay Area because it was the first region in the USA to implement school closures, and has continued to maintain closures as of February 2021 [30].

## 2. Material and methods

We conducted a survey to ascertain the contact rates of children and their adult family members during spring school closures. We used these contact rates within an individual-based transmission model to examine the impact of spring school closures and reopening strategies.

### 2.1. Survey methodology

We implemented a social contact survey of school-aged children in nine Bay Area counties (Alameda, Contra Costa, Marin, Napa, San Francisco, San Mateo, Santa Clara, Solano, Sonoma) during county-wide shelter-in-place orders. Survey respondents reported the number and location of non-household contacts made within six age categories (0–4, 5–12, 13–17, 18–39, 40–64 and 65+ years) throughout the day prior. A contact was defined as an interaction within 2 metres lasting over 5 seconds.

Eligible households contained at least one school-aged child (pre-kindergarten to grade 12 (around 17 or 18 years old)). A first sample was obtained using a web-based contact diary distributed in English via social networks (Nextdoor, Berkeley Parents Network) between 4 May and 1 June 2020. A second sample was procured between 18 May and 1 June 2020 via an online panel provider (Qualtrics) to be representative of Bay Area race/ethnicity and income. In both samples, surveys asked one adult respondent per household to respond on their behalf and for all children in their household. The survey also recorded household demographic information, including adult occupation status. A copy of the survey tool is included in the electronic supplementary material.

### 2.2. Survey analysis

To adjust for potential selection bias, we calculated post-stratification weights reflecting joint distributions of race/ethnicity and income of the counties' combined population using the 2018 1-year American Community Survey Public Use Microdata Sample from the nine counties. To account for potential bias due to occasional non-response on location questions, we applied a second set of weights equal to the inverse of the probability of response, conditional on race and income (fixed effect) and household ID (random effect). Weighted and unweighted survey data yielded similar results (electronic supplementary material, figure S2).

Contact matrices generated using weighted and unweighted survey data were stratified by income, race and location of contact. To determine whether an individual's total reported contacts varied by key covariates, we fitted a multivariable linear regression model accounting for a household random effect and fixed effects for age, race, household income, number of household members, single parent household, weekday of reported contact and school type, and a binary indicator of whether more adults within the household worked at home during shelter-in-place than before shelter-in-place.

We conducted all statistical analyses using R (v. 3.2.2; R Foundation for Statistical Computing, Vienna, Austria), and fitted random effects models using the *lme4* package [31].

### 2.3. Transmission model

Using survey-derived estimates of contact patterns, we developed a transmission model to estimate the number of cases, hospitalizations and deaths that would have occurred under various counterfactual intervention scenarios (e.g. if schools had remained open), and used this model to simulate the impact of various school reopening strategies.

First, we generated 1000 synthetic populations representative of the demographic composition of Oakland, California, following previous methods (electronic supplementary material) [6]. Each individual was assigned an age, household and occupation status (student, teacher, school staff, other employment, not employed), upon which membership in a class or workplace was based. Each individual represented 25 individuals in the real population. All possible pairings of individuals were partitioned into one of six types of interactions, according to a hierarchy of highest shared membership: household > classroom or workplace > grade > school > community [32]. Community interaction represented the number of contacts expected between individuals from age groups *i* and *j* scaled by the total number of individuals in age group *j*, such that the total number of contacts per agent stayed constant were the simulated population to be scaled up. We separated schools into elementary (grades K–5), middle (grades 6–8) and high (grades 9–12) schools.

**Figure 1.**
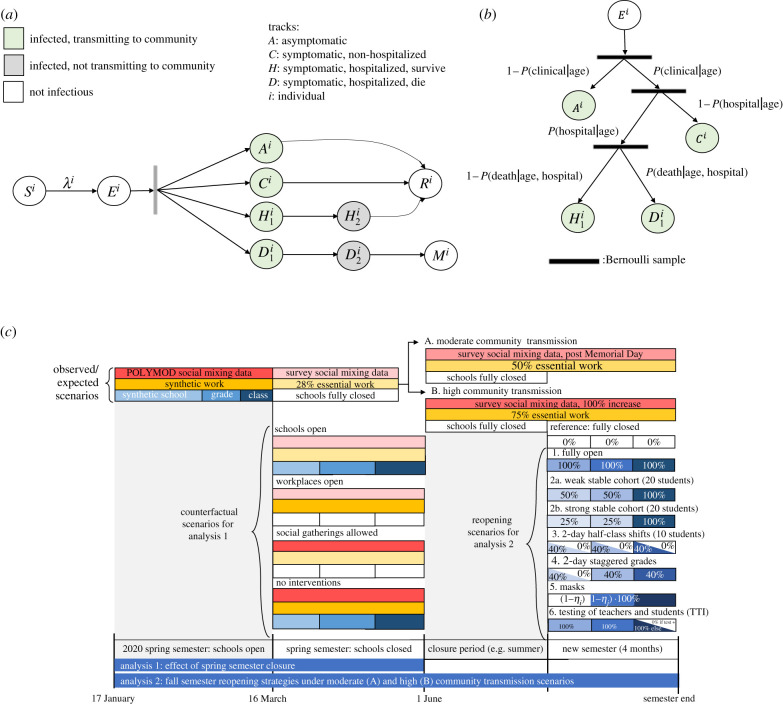
Model schematic (*a*) Schematic of the agent-based susceptible–exposed–infected–recovered (SEIR) model. *S*, susceptible; *E*, exposed; *A*, asymptomatic; *C*, symptomatic, will recover; *H*_1_, symptomatic and will recover, not yet hospitalized; *H*_2_, hospitalized and will recover; *D*_1_, symptomatic, not yet hospitalized; *D*_2_, hospitalized and will die; *R*, recovered; *M*, dead; *λ*, force of infection defining movement from *S* to *E*. Superscript *i* refers to individual. After an agent enters the exposed class, they enter along their predetermined track, with waiting times between stage progression drawn from a Weibull distribution. (*b*) Schematic of the conditional probabilities by which agents are assigned a predetermined track. (*c*) Schematic of interventions simulated in the SEIR model. The first analysis examines transmission between 17 January and 1 June, and tests the effect of several counterfactual scenarios that took place between the enactment of shelter-in-place (16 March) and the original end of the spring semester (1 June). The second analysis examines transmission over a subsequent four-month semester, and tests the effect of several simulated reopening strategies for the semester, expected to occur under a high and moderate community transmission scenario. Boxes represent categories of social contacts, including community (red), work (yellow), school (light blue), grade (medium blue) and classroom (dark blue). Percentages in the boxes represent the percentage of the contact rate experienced under a given intervention or counterfactual scenario (e.g. 0% represents a full closure).

We then developed a discrete-time, age-structured, individual-based stochastic model to simulate SARS-CoV-2 transmission dynamics in the synthetic population (figure 1*a*). At each time increment, representative of 1 day, each individual is associated with an epidemiological state: susceptible (*S*), exposed (*E*), asymptomatic (*A*), symptomatic with non-severe illness (*C*), symptomatic with severe illness (*H*_1_, *D*_1_) resulting in eventual hospitalization before recovery (*H*_2_) or hospitalization before death (*D*_2_), recovery (*R*) or death (*M*). A full description of the transmission model methodology is provided in the electronic supplementary material.

Based on their type of interaction (e.g. household, class, community), the daily contact rate between individuals *i* and *j* on day *t*, $K_{ij,t}$, was estimated for pairs of individuals following a previous study [32]. Contact rates were scaled by a time-dependent factor between 0 (complete closure) and 1 (no intervention), representing a social distancing intervention to reduce contact between individual pairs. Pairs with a school or workplace interaction were reassigned as community interactions under closures. Because symptomatic individuals mix less with the community [33], we incorporated isolation of symptomatic individuals and quarantine of their household members. Following prior work, we simulated a 100% reduction in daily school or work contacts and a 75% reduction in community contacts for a proportion of symptomatic individuals, and an additional proportion of their household members [34]. This means that a proportion of students and staff would stay home from school if they themselves were symptomatic, while a smaller percentage would stay home from school if one of their household members was symptomatic. We assumed that individuals were in the infectious class for up to 3 days prior to observing symptoms [35], during which time they did not reduce their daily contacts.

To parameterize the model, we calculated the mean transmission rate of the pathogen, $\bar{\;\beta }$, using the next-generation matrix method [36]. Briefly, assuming an initial *R*_0_ of 2.5 [37,38], we solved for $\bar{\;\beta }$ as the ratio between *R*_0_ and the product of the infection duration and the weighted mean number of daily contacts per individual during the pre-intervention period (electronic supplementary material, equation 2). To represent age-varying susceptibility [39], we then calculated an age-stratified $\beta_{i}$, which incorporated varying relative susceptibility by age while permitting the population mean to be $\bar{\;\beta }$ (electronic supplementary material, equations 3–4). Owing to uncertainty in the relative susceptibility of children to SARS-CoV-2 infection compared with adults [21], we modelled scenarios where children under 10 years were half as susceptible as older children and adults, children under 20 years were half as susceptible as adults, and all individuals were equally as susceptible (see the electronic supplementary material for a tabular review of studies on age-dependent susceptibility). Using these methods, we calculated the secondary attack rate among household members to be between 9.6% and 11.1%, in agreement with prior studies [23,40–42].

Transmission was implemented probabilistically for contacts between susceptible (*S*) and infectious individuals in the asymptomatic (*A*) or symptomatic and non-hospitalized states (*C*, *H*_1_, *D*_1_). Movement of individual *i* on day *t* from a susceptible to exposed class is determined by a Bernoulli random draw with probability of success given by the force of infection, $\lambda_{i,t}$,

1 $$\lambda_{i,t}=\alpha \beta_{i}\overset{N}{\underset{\;j=1}{\sum }}K_{ij,t}A_{\;j,t}\;+\beta_{i}\overset{N}{\underset{\;j=1}{\sum }}K_{ij,t}(C_{\;j,t}+{H_{1}}_{\;j,t}+{D_{1}}_{\;j,t})\;,\;$$

where *N* is the number of individuals in the synthetic population (*N* = 16 000) and α is the ratio of the transmissibility of asymptomatic individuals to symptomatic individuals. Using estimates from studies evaluating risk of symptoms by age [39], we assumed that 21% of infected individuals less than 20 years and 69% of infected individuals 20 years and older experienced symptoms [39]. Following previous work [39], we assumed α to be less than 1, as asymptomatic individuals may be less likely to transmit infectious droplets by sneezing or coughing [43]. We explored differences in age-dependent transmissibility by modelling scenarios that varied α.

Whether an individual remained asymptomatic or was hospitalized or died was determined via Bernoulli random draws from age-stratified conditional probabilities (figure 1*b*; electronic supplementary material, table S5). The durations of time spent in each disease stage were sampled from Weibull distributions (electronic supplementary material, table S5). Simulations were initiated on 17 January two weeks before the first known case [44], assuming a fully susceptible population seeded with a random number (range: 5–10) of exposed individuals. We averaged results over 1000 independent realizations, using one random draw from the synthetic population, and estimated confidence intervals as the 2.5th and 97.5th percentile of all realizations.

### 2.4. Modelled contact rates and interventions

A shelter-in-place order was announced for Bay Area counties on 16 March 2020 [30], following which 28% of work continued in-person [45], and schools were closed. Between 17 January and 16 March, transmission was simulated as described above, deriving community contact rates during typical conditions using data from the POLYMOD study in the UK [46].

We then simulated transmission during 17 March–1 June, the remainder of the spring semester in the 2019–2020 academic year (figure 1*c*), first under real-world conditions: no school contacts, 28% workforce participation [45] and community contacts derived from our social contact survey. Modelled output matched well with available data on hospitalizations, deaths and seroprevalence (electronic supplementary material, figure S5). We then simulated transmission under counterfactual scenarios where: (i) schools remained open; (ii) workplaces remained open; and (iii) non-essential community contacts continued.

Community contact matrices were derived for each intervention based on survey and POLYMOD data to account for differences in location-specific contacts (e.g. transportation contacts increase for in-person work, daycare contacts decrease when school is in session) (electronic supplementary material, figure S2 and table S4). For all counterfactual scenarios, except those permitting non-essential community contacts, we assumed 50% of household members of symptomatic cases reduced their community contacts by 75% and their work or school contacts by 100% [34]. We estimated the number of cases, hospitalizations and deaths averted by the intervention as the difference between these outcomes for the counterfactual scenarios minus the modelled real-world scenario.

Lastly, we simulated the effect of school reopening strategies over a subsequent four-month semester (figure 1*c*). We established initial conditions for these simulations by initiating model runs spanning a school-closure period, and then modelled the effect of reopening strategies under two susceptibility assumptions (children less than 20 years half versus equally as susceptible as adults) and two transmission contexts (high and moderate community transmission). The high transmission context is characterized by 75% of workplaces remaining open and non-essential community contacts double what we observed in our survey; the moderate transmission context is characterized by 50% of workplaces remaining open and non-essential community contacts equal to that observed in our survey after Memorial Day (25 May 2020). In our simulations, the school-closure period aligned with the summer break, and the reopening period with the 2020 fall (autumn) semester; however, Bay Area school districts remained closed throughout the duration of the fall 2020 semester. We thus model various transmission scenarios in the school-closure period so as to enable model simulations for the new semester to be generalizable to either a fall or a spring semester reopening after a variable closure period.

We simulated six school reopening strategies (figure 1*c*; see the electronic supplementary material for details): (1) schools open without precautions; (2) classroom groups are enforced, reducing other grade and school contacts by (a) 50% (weak cohort) or (b) 75% (strong cohort); (3) hybrid with class sizes halved, and each half attends two staggered days each week; (4) hybrid with class sizes maintained, and half the school attends two staggered days each week according to grade groups; (5) all students and faculty wear masks; (6) faculty and/or students are tested with 85% sensitivity on a (a) weekly or (b) monthly basis [47], with positive cases isolated and their class quarantined for 14 days (periodic test–trace–isolate, TTI). We examined the six interventions by themselves and in combination (e.g. cohorts, masks and TTI). The average class size was 20 students.

Masks were assumed to reduce both outward and inward transmission by *η_i_* [48], where *η_i_* represents the efficacy of the mask for individual *i*. Meta-analyses that included cotton masks worn by the general population found a reduction in infection risk of about 50% to the adult wearer [49]. Mask efficacy is lower among children than among adults, and lower in younger children (about 15%) than in older children, possibly related to inferior fit or compliance with continuous use [50,51]. We therefore assumed age-dependent mask efficacy (15% for elementary students, 25% for middle school students, 35% for high school students, 50% for teachers/staff). We estimate excess infections (symptomatic only and all infections), hospitalizations and deaths attributable to school-based transmission as the cumulative incidence of infections, hospitalizations and deaths under each school reopening scenario minus the cumulative incidence under a school-closure scenario. We then identified which set of interventions is needed to reduce excess risk of symptomatic illness for teachers (the sub-population determined to be at highest risk) such that less than one additional per cent becomes infected.

## 3. Results

### 3.1. Contact patterns

**Figure 2.**
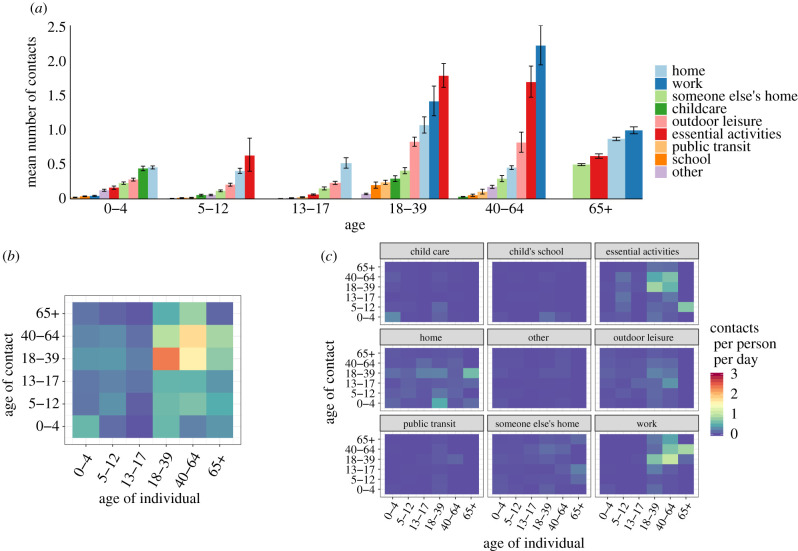
Social contact patterns between children and adult family members of Bay Area households, 4 May–1 June 2020. (*a*) Average daily contacts per age group at nine pre-specified locations. (*b*) Average daily contacts per person by age category of the survey respondent and reported contact, unweighted. (*c*) Average daily contacts per person at each of the nine locations. Panels (*b,c*) share a legend.

Six hundred and twelve households provided contact histories on behalf of 819 school-aged children in the Bay Area (electronic supplementary material, table S1). The majority of non-household contacts occurred between individuals in the same age category, and while performing essential activities (such as grocery shopping, laundering clothing or receiving healthcare), at work, home or during an outdoor leisure activity (figure 2*a,c*). Children aged 5–12 years had twice as many non-household contacts (1.58 contacts per child per day) as teenagers aged 13–17 years (0.78 contacts per teenager per day) (figure 2*b*).

In multivariable models adjusting for demographic and household characteristics, households identifying as Hispanic or Latinx had 2.32 (95% CI: 0.08–4.50) more contacts on average than non-Hispanic or Latinx households (table 1). Households that did not indicate an increase in the number of adults working from home during shelter-in-place compared with before shelter-in-place had 1.85 (95% CI: 0.16–3.52) more contacts than households with more adults working at home during shelter-in-place.

**Table 1.** Differences in social contacts by demographic variables. Coefficients from a multivariable linear mixed model adjusted for race (reference: white alone), self-reported household income (reference: <US$150 000), whether household identified as Hispanic (reference: not Hispanic), whether household was a single parent household (reference: multi-parent household), whether date of reported contacts were weekend or weekday (reference: weekday), whether child attended a public or non-public school (including private, charter, home, school or other), age of individual in years, whether the date of reported contacts occurred over the Memorial Day weekend (24 May–26 May 2020, reference: not the holiday weekend), and the change in number of adults working at home during shelter-in-place (SIP) (reference: more adults working at home during SIP).

	average adjusted difference in daily contact rate (95% CI)
race (*ref: white alone)*	
Asian alone	−0.77 (−2.4, 0.89)
black or African American alone	−1.33 (−3.93, 1.35)
other race alone	−2.94 (−6.46, 0.69)
two or more races	−1.43 (−4.66, 1.72)
Hispanic household	2.32 (0.08, 4.5)
household income >US$150 K	−0.35 (−1.8, 1.12)
no. individuals in household	0.25 (−0.59, 1.05)
single parent household	−0.32 (−3.73, 3.13)
weekend	1.63 (−0.45, 3.69)
public school	−0.2 (−1.79, 1.41)
age	0.0 (−0.16, 0.16)
Memorial Day weekend	1.28 (−1.03, 3.62)
less or same no. adults working from home during SIP	1.85 (0.16, 3.52)

### 3.2. Impact of spring 2020 school-closure policies

#### 3.2.1. Assuming children less than 10 years are half as susceptible to SARS-CoV-2 infection and asymptomatic individuals have lower transmissibility

**Figure 3.**
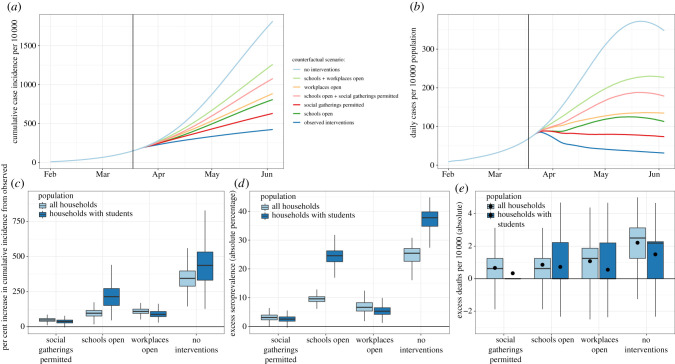
Effect of spring semester interventions. We simulated transmission between 17 February and 1 June assuming children less than 10 years are half as susceptible to infection as older children and adults. Between 16 March (enactment of shelter-in-place orders) and 1 June (the end of the spring school semester), we assessed potential outcomes under various counterfactual scenarios: (1) schools had remained open for the remainder of the school semester; (2) workplaces had remained open; (3) social gatherings were permitted; (4) no interventions were enacted. (*a*) Modelled cumulative incidence according to the counterfactual scenario examined. Modelled predictions are not adjusted for under-reporting, which is expected to be substantial. (*b*) Daily incidence per 10 000 per counterfactual scenario examined. (*c*) The per cent increase in cumulative incidence from observed incidence between 17 February and 1 June, stratified by counterfactual scenario and population sub-group. (*d*) The absolute difference in the per cent of the population seropositive for each counterfactual scenario compared with the modelled, observed seroprevalence between 17 February and 1 June, stratified by population sub-group. (*e*) The per cent increase in deaths per 10 000 from observed between 17 February and 1 June, stratified by counterfactual scenario and population sub-group. The distribution of estimated death rate across 1000 realizations was skewed, so black dots representing the mean number of excess deaths per 10 000 are added.

As of 1 June, the nine Bay Area counties had reported 14 202 cases of COVID-19 [52]. Assuming a ratio (*α*) of the transmissibility of asymptomatic individuals to symptomatic individuals of 0.5, and susceptibility of children under 10 years set to half that of older children and adults, we estimated that there would have been 1.98 (95% CI: 0.44, 2.6) times more cases of COVID-19 throughout the nine Bay Area counties between 16 March and 1 June than observed had all K–12 schools remained open (figure 3), corresponding to 13 842 (95% CI: 6290, 23.040) excess confirmed cases. We estimated 3.16 (95% CI: 1.79, 4.89) times more cases would have occurred among families of students in grades K–12 than observed. Examining cases averted by school-level closures, we estimated that, if elementary schools alone had remained open, the Bay Area would have recorded 2167 additional cases (95% CI: −985, 5572), while if only middle schools had remained open an additional 5884 cases (95% CI: 1478, 11 550) would have been observed, and if high schools alone had remained open an additional 8650 cases would have been observed (95% CI: 3054, 15 940). An additional 6370 (95% CI: 1853, 12 122) cases would have been recorded if middle schools and elementary schools had remained open. This means that when one level of schooling is closed, each additional closure has a smaller marginal benefit. This is in part driven by households with multiple school-aged children, who share the same household contacts to whom an infection acquired within school could spread (electronic supplementary material, figure S6a).

By comparison, had all workplaces remained open, we estimated that, as of 1 June, there would have been 15 813 additional confirmed cases (95% CI: 9963, 22 617), reflecting 2.11 (95% CI: 1.70, 2.59) times more cases than observed. If non-essential outings and social gatherings had been permitted, we estimated that there would have been an additional 7030 (95% CI: 3118, 11 676) confirmed cases, reflecting 1.50 (95% CI: 1.22, 1.82) times more cases than observed. All three interventions together helped avert an estimated 49 023 confirmed cases. The excess cases associated with opening both workplaces and schools was additive (electronic supplementary material, figure S6b). The effects of limiting social gatherings depended upon whether there were concurrent workplace or school closures; the number of excess cases associated with allowing social gatherings and in-person work, or allowing social gatherings and in-person school, was higher than the excess cases associated with either individually. This suggests that, by itself, social distancing is the least effective intervention; yet it becomes an important control measure when workplaces or schools are open. Reopening a school or workplace raises an individual's exposure to infection, which then increases the risk of a social gathering of individuals from multiple schools or workplaces, while also permitting infections to jump workplaces or schools (electronic supplementary material).

We find that both school and workplace closures in the spring of 2020 were necessary to achieve a sustained *R* < 1. We estimated that the highest COVID-19 hospitalization occupancy that would have been observed on any one day during shelter-in-place if schools were open was 10.6 (95% CI: 6.0, 16.0) per 10 000 population, representing an excess of 4.42 individuals per 10 000 from the modelled real-world hospitalization occupancy. As the Bay Area has, on average, 12.3 beds available per 10 000 population (22 beds per 10 000 capacity at 56% non-occupancy rate) [53], school closures permitted over a third of available beds to remain available, but were not necessary to keep Bay Area healthcare systems under capacity. As of 1 June 2020, the Bay Area had 3997 confirmed deaths from COVID-19 [52]. We estimate that school closures averted 0.63 deaths (95% CI: −1.25, 3.75) per 10 000 population, corresponding to 663 averted deaths across the Bay Area, fewer than workplace closures (estimated 828 deaths averted) and more than restrictions on social gatherings (estimated 503 deaths averted).

#### 3.2.2. Assuming individuals less than 20 years are half as susceptible to SARS-CoV-2 and asymptomatic individuals have lower transmissibility

**Figure 4.**
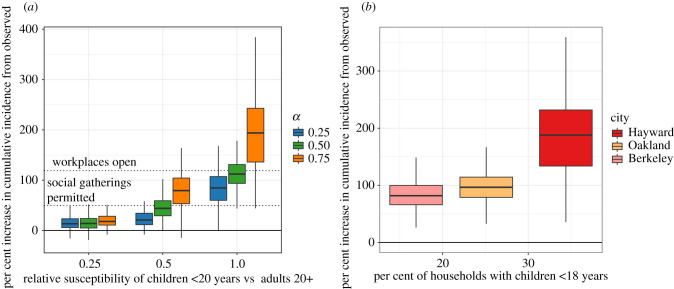
Influence of key epidemiological parameters on the effectiveness of school closures. The per cent increase in cumulative incidence from observed incidence over the period 17 February–1 June had schools remained open between 17 March and 1 June. (*a*) Results are reported for modelling scenarios that varied the ratio of the susceptibility of individuals under 20 years to adults 20 or older, and the ratio of the force of infection for asymptomatic infections to symptomatic infections (*α*). Dashed lines indicate the per cent increase in incidence from observed that would have been expected if workplaces had remained open, and if social gatherings were permitted. (*b*) Results are reported for synthetic populations with varying levels of the proportion of households with children under 18 years of age, reflecting three major Bay Area cities (Berkeley, Oakland, Hayward), assuming children under 10 are half as susceptible as older children and adults.

The estimated impact of school closures in spring 2020 strongly depended on the relative susceptibility of children to adults (figure 4*a*). Under the assumption that all individuals under 20 years are half as susceptible to SARS-CoV-2 compared with adults, school closures would be the least effective intervention when compared with workplace and social distancing strategies, avoiding an estimated 4179 cases (95% CI: 308, 10 583) and 202 deaths (0.26 deaths per 10 000 population, 95% CI: −1.25, 2.50) between 17 March and 1 June across the Bay Area.

#### 3.2.3. Assuming equal susceptibility to infection across all ages and asymptomatic individuals have lower transmissibility

Under the assumption of equal susceptibility to infection among all ages, the estimated impact of school closures quadrupled, from 4179 averted cases to 16 348 (95% CI: 8325, 25 363) averted cases, making school closures the most effective intervention. Likewise, with equal susceptibility across ages, the estimated number of deaths averted by school closures in the nine Bay Area counties between 17 March and 1 June more than tripled, from 202 to 655 averted deaths, corresponding to an excess death rate of 0.84 (−1.25, 3.13) per 10 000 population. The excess death rate averted by workplace closures was only slightly higher, at 0.90 excess deaths per 10 000 (95% CO: −1.25, 3.13) between 17 March and 1 June.

#### 3.2.4. Influence of transmissibility of asymptomatic individuals and household composition

At low levels of susceptibility (i.e. one-quarter that of adults) among children, the impact of school closures was small, and the ratio of transmissibility of asymptomatic individuals to symptomatic individuals (*α*) had little influence on the impact of spring school closure policies (figure 4*a*). As children increase in susceptibility relative to adults, the influence of *α* becomes more pronounced (figure 4*a*).

We found a significant positive relationship between the number of cases averted by school closures and the proportion of households in the population with children under 18 years (figure 4*b*). For each 1% increase in the proportion of total households that have children under 18, we estimate an additional 5.8% increase over observed incidence had schools remained open throughout the spring semester.

### 3.3. Simulated impact of reopening strategies

**Figure 5.**
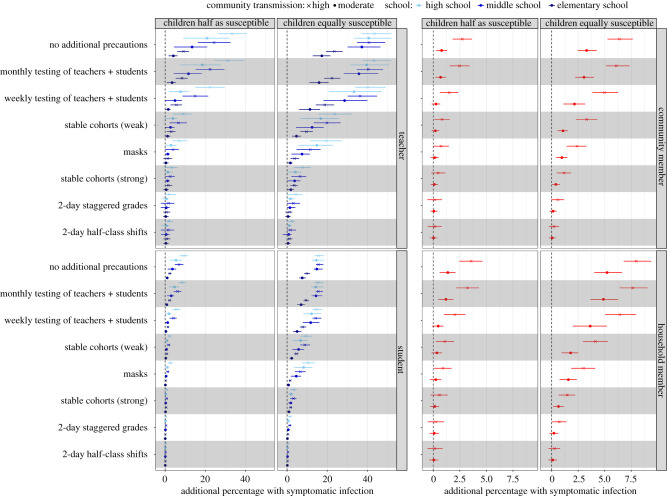
Excess risk by sub-group associated with school reopening strategies over a subsequent four-month semester. Points and horizontal lines show the additional proportion (mean and interquartile range) of each sub-group expected to experience clinical infection over the course of a four-month semester compared with if schools were closed under each reopening scenario and the four transmission contexts: children half and equally as susceptible as adults crossed with moderate and high community transmission. Colours indicate the transmission across levels of schooling (elementary, middle and high) while the shape of the mean point indicates the level of community transmission (circle, moderate; cross, high). ‘Teachers' includes teachers and all other school staff.

The estimated risk of symptomatic infection associated with reopening for a subsequent four-month semester—across moderate to high transmission contexts—is highest for teachers and other school staff, followed by students and other household members of students and teachers/staff (figure 5). Owing to larger average school sizes, we found high schools were at higher risk, followed by middle schools, then elementary schools. Staggered 2-day school weeks with halved class sizes provided the largest reduction in risk among all interventions considered, followed by strong stable cohorts of class groups, then wearing face masks. In the absence of other interventions, periodic (tests administered weekly or monthly) TTI strategies have low effectiveness, but when combined with strict social distancing measures a modest reduction in community cases was possible as infectious individuals and their contacts identified in the school environment were quarantined (i.e. have their community contacts reduced by 75% for 14 days). Excess seroprevalence, hospitalizations and deaths associated with school reopening, as they varied with respect to differing assumptions about child susceptibility and community controls, are detailed in electronic supplementary material, tables S6–S9.

#### 3.3.1. Assuming individuals less than 20 years are half as susceptible

**Figure 6.**
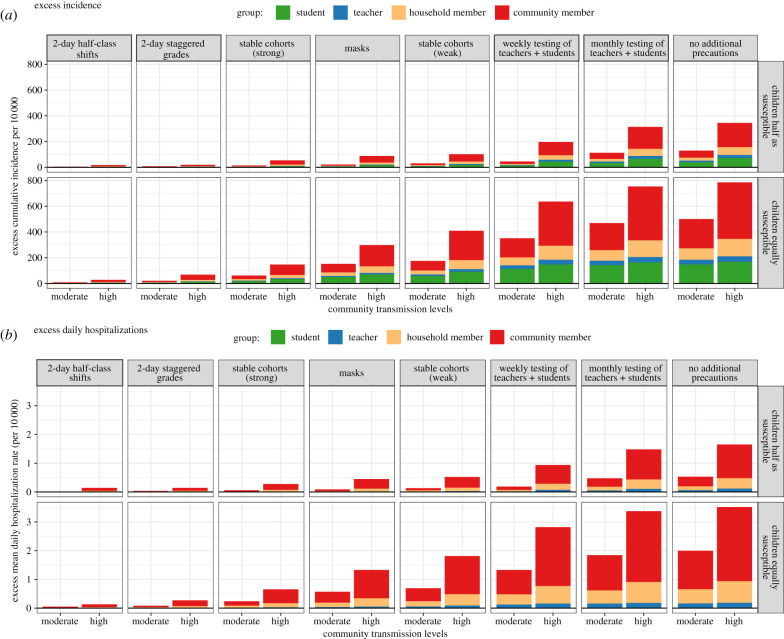
Population-level excess incidence and hospitalizations associated with reopening strategies over a four-month semester. Excess cumulative incidence per 10 000 (*a*) and excess daily hospitalization, on average, per 10 000 (*b*) that would be expected over a four-month semester for each reopening strategy compared with if schools were closed. Bars are stratified by the moderate and high community transmission scenario and coloured according to the sub-group contributing cases. ‘Teachers' includes teachers and all other school staff.

We examined the effect of school reopening when modest community controls (e.g. 50% in-person work and continued social distancing) were in place, leading to moderate community transmission. With no precautions taken within school settings, we estimated that an additional 21.0% (95% CI: 0, 46.0%) of high school teachers, 13.4% (95% CI: −2.2, 38.6%) of middle school teachers and 4.1% (95% CI: −1.7, 12.0%) of elementary school teachers would experience symptomatic illness over the four-month reopening period, compared with expectations if schools were closed (figure 5). We estimated that the daily hospitalization occupancy rate would increase by an average of 0.53 (95% CI: −0.58, 1.73) hospitalizations per 10 000 individuals (roughly 4.2% of Bay Area available bed capacity), of which 0.13 (95% CI: −0.29, 0.58) and 0.33 (95% CI: −0.58, 1.30) hospitalizations per 10 000 would be among household members of students and other community members, respectively (figure 6*b*). We estimated an excess total death rate of 0.56 (95% CI: −1.88, 3.13) per 10 000 over the four-month period, corresponding to 434 (95% CI: −1451, 2418) deaths across the Bay Area, of which 287 would be among community members without students in their household, 114 among household members of students, 31 among teachers and one among students.

We also examined the effect of reopening when lessened community controls (e.g. 75% in-person work and limited social distancing) were in place, leading to high community transmission. With no precautions taken within school settings, we estimated that an additional 33.3% (95% CI: 11.1, 53.6%) of high school teachers, 24.4% (95% CI: 4.3, 44.4%) of middle school teachers and 9.1% (95% CI: 0.9, 20.0%) of elementary school teachers would experience symptomatic illness (figure 5). We estimated that the daily hospitalization occupancy rate would increase by an average of 1.65 (95% CI: −0.17, 3.38) hospitalizations per 10 000 individuals, of which 0.37 (95% CI: −0.22, 1.01) and 1.17 (95% CI: −0.36, 2.70) per 10 000 would be among household members of students or teachers and other community members, respectively (figure 6*b*). We estimated an excess total death rate of 1.73 (95% CI: −2.50, 6.25) per 10 000, corresponding to 1341 (95% CI: −1934, 4837) deaths across the Bay Area, of which 1026 would be among community members, 254 among household members, 60 among teachers and one among students.

At moderate community transmission, we estimated that reducing excess risk of symptomatic illness for teachers to less than 1% would require either strict adherence to staggered school weeks (either as half classes or grades) or a combination of stable cohorts (weak or strong), wearing face masks and monthly TTI (table 2, which also details interventions necessary in high transmission contexts). Strong stable cohorts, 2-day staggered grades or strong stable cohorts combined with wearing masks and periodic TTI protocols are associated with reductions in deaths of 85%, 95% and 95%, respectively.

**Table 2.** School-based interventions to reduce risk. This table colours the reopening strategies examined by whether or not they are sufficient to reduce the additional proportion of teachers and other school staff experiencing symptomatic illness across a four-month semester to less than 1% of teachers. Strategies coloured in green are strategies which reduce the excess number of teachers with symptomatic illness to less than 1%. Strategies coloured in grey are strategies which do not reduce the excess number of teachers with symptomatic illness to less than 1%. Results are stratified by high school and elementary school teachers.

community transmission:	elementary school		high school	
	moderate	high	moderate	high
**children half as susceptible**				
stable cohorts (weak)				
masks				
stable cohorts (strong)				
2-day staggered grades				
2-day half-class shifts				
stable cohorts^a^, masks + monthly TTI				
2-day staggered grades + stable cohorts^a^				
2-day half-classes + stable cohorts^a^				
all interventions^b^				
**children equally as susceptible**				
stable cohorts (weak)				
masks				
stable cohorts (strong)				
2-day staggered grades				
2-day half-class shifts				
stable cohorts^a^, masks + monthly TTI				
2-day staggered grades + stable cohorts^a^				
2-day half-classes + stable cohorts^a^				
all interventions^b^				

^a^Weak or strong.

^b^All interventions include: masks, staggered grades, stable cohorts and monthly TTI.

TTI, test–trace–isolate.

We found that reducing community transmission via enhanced community controls significantly reduced the excess risk to teachers across all grades, from 18.4% (95% CI: 7.7, 27.9%) to 10.3% (95% CI: 0.4, 20.7%) in the no precaution scenario, with the influence of community transmission levels minimized as school-based interventions became stronger. Under minimal within-school interventions, the level of community transmission strongly determined whether the effect of school reopenings would be associated with increased incidence among the general community (non-students, teachers or family members). In high transmission settings where schools open without precautions, we estimated that the majority (59%) of the excess cases would be among community members, whereas in moderate transmission settings fewer than half (45%) of the excess cases would be among community members (figure 6*a*).

#### 3.3.2. Assuming equal susceptibility across all ages

In scenarios evaluating both moderate and high community transmission, when susceptibility to infection is assumed constant across all ages, we estimated a higher proportion of additional clinical infections among all sub-populations and reopening strategies than in the reopening scenario where children were half as susceptible (figure 5). Notably, if no precautions are taken within school settings, at moderate levels of community transmission, we estimated nearly four times as many elementary school teachers would experience additional clinical infections if children are equally susceptible (17.3%, 95% CI: 4.4, 30.0%) as the equivalent scenario where children are half as susceptible (4.1%, 95% CI: −1.7, 12.0). Similarly, over three times as many middle school teachers (37.2%, 95% CI: 4.6, 58.1% versus 13.4%, 95% CI: −2.2, 38.6%) and nearly two times as many high school teachers (40.7%, 95% CI: 1.9, 61.1% versus 21.0%, 95% CI: 0, 46.0%) would experience symptomatic illness when comparing the relative susceptibility of children at moderate levels of community transmission if no additional precautions are taken in school settings. At moderate levels of community transmission, increasing the relative susceptibility of children to adults also quadrupled the excess daily hospitalization occupancy rate in moderate transmission scenarios from 0.53 hospitalizations per 10 000 individuals when children are half as susceptible to 2.00 (95% CI: 0.36, 3.67) hospitalizations per 10 000 individuals if children are equally susceptible, leading to more than four times the number of absolute deaths among community members (287 community member deaths if children are half as susceptible versus 1159 community member deaths if children are equally as susceptible) (figure 6*b*).

Regardless of the relative susceptibility of children to adults, across both moderate and high community transmission settings, a strict adherence to a combination of within-school distancing interventions (e.g. combining staggered half-classes or staggered grades with stable cohorts; combining stable cohorts with wearing face masks and monthly TTI protocols) was required to reduce the excess risk of symptomatic illness for high school teachers and all other school staff to less than 1% (table 2). The benefit of having a strong (75%) versus a weak (50%) reduction in non-classroom (non-cohort) contacts is most notable when children are highly susceptible. For instance, in a high transmission context, reducing non-classroom contacts by 50% and 75% lowers the excess risk to all teachers from 32.1% to 15.3% and 5.3%, respectively. If children are half as susceptible, the excess risk to all teachers is lowered from 18.4% to 5.2% and 3.4%, respectively (figure 5).

## 4. Discussion

Gaps in our understanding of contact patterns among US schoolchildren have limited previous efforts to estimate the effect of school closures on COVID-19 transmission in a community of demographically heterogeneous households. We found evidence of a higher average community contact rate among lower income and Hispanic children during shelter-in-place orders, consistent with literature demonstrating limited ability of low-income communities to shelter-in-place [54], which contributes to the disproportionately high incidence and mortality rates among low-income or Hispanic communities [55]. Differences in total contacts between Hispanic and non-Hispanic respondents were driven by working-aged adults (18–65 years) and young children (0–12 years). As Hispanic individuals make up a disproportionate number of essential workers in the Bay Area [45], these findings may reflect both contacts at work and childcare. Indeed, while our survey found higher contact rates in elementary students than in high school students, social mixing data during non-epidemic periods report higher community contact rates among high school students [46]. Elementary students may have more limited ability to shelter-in-place than high school students because of accompanying family members during essential activities and requiring daycare.

In the 17 March–1 June spring 2020 semester period, we estimated that school closures averted 13 842 confirmed cases and 663 deaths in the Bay Area. Under the lowest risk scenario examined, we found that reopening for a four-month semester without any precautions would increase risk for students (an additional 3.0% of students across all grade levels infected over the four-month reopening period), family members of students (an additional 1.4% infected) and especially teachers/staff (an additional 10.3% across all grade levels). Our results are consistent with other models that project large increases in transmission owing to in-person schooling conducted with no safety measures, with substantial reductions in school-attributable transmission possible when within-school and community intervention measures are in place [13,14,16,56,57]. Our results are also consistent with empirical evidence showing high transmission among a summer camp where children interacted in large cohorts [58], high seroprevalence among teachers and students from a high school setting with limited safety measures [28], moderate transmission among teachers from schools with rare face mask use and some social distancing [29,59] and low transmission in schools that adopted a cohort or hybrid system, masks or TTI protocols [17,60].

Some reopening strategies can result in few in-school transmissions among students and teachers alike, according to our findings. Most notably, our model found that reducing in-school mixing via classroom cohorts or hybrid scheduling is an effective means of reducing the risk of school-attributable illness across all levels of education, especially when combined with universal masking. These findings concur with observations of schools that reopened with universal masking, social distancing and a hybrid or cohort approach and avoided large outbreaks [15,17,60]. While we find that high community transmission increases the risk of within-school transmission across all measures studied, the influence of community transmission is minimized as the effectiveness of the within-school measures increases. Our findings therefore support the most recent US Centers for Disease Control and Prevention guidance, which states that community transmission rates are important to monitor when planning for the reopening of schools, but the essential elements for reopening are implementation of within-school measures—masks, physical distancing, handwashing and contact tracing—with priority given to masks and distancing [61]. We found that if the prioritized essential elements of masks and physical distancing via a cohort or hybrid system are not met, outbreaks are plausible. Under such scenarios with minimal within-school interventions, community interventions (e.g. workplace closures and reductions in social gatherings) play a larger role in moderating within-school transmission. This is consistent with outbreaks documented in childcare settings that lack safety precautions [12,58,62] and with reports from the UK that the risk of outbreaks in schools without mask requirements increased with community transmission levels [19,29]. We found that teachers and staff would bear a disproportionate burden of infection if an outbreak occurred, in agreement with available data on school transmission [29,59]. It is thus essential to ensure that specific precautions are available to support this population, including safe spaces for lunch breaks, virtual faculty meetings and financial and logistical support if quarantine is needed.

We find that reducing the risk of school-attributable illness to below 1% in each population sub-group is most feasible in elementary schools (using, for instance, masks and stable cohorts). Achieving the same protection within high schools, by comparison, would require combining and maintaining two or more strict social-distancing interventions, such as staggered 2-day school weeks, wearing masks and stable cohorts, which may present a challenge as high school students often interact with several different classroom groups across a single school day. However, a staggered school schedule is likely to be more feasible for high school families, as teenagers may be more amenable to self-remote instruction. The idea that elementary schools pose a lower transmission risk than high schools is widely supported [2], both from modelling studies [13,57,63] and empirically [27,28,62]. For example, high school environments have larger student, teacher and staff populations. Even if younger children are as susceptible as older children, we estimate that reopening high schools without precautions yields an estimated three to five times more risk of symptomatic infection to teachers/staff than reopening of elementary schools, depending on the level of community transmission. If susceptibility increases with age, as some evidence suggests [21,39,64], we estimated that high school teachers may experience as much as 5–10 times greater risk of symptomatic infection than elementary school teachers, depending on the level of community transmission. These findings agree with empirical data from Sweden, which found that risk to teachers increased with student age [59].

The age-structured contact rates from the Bay Area are similar to those captured from households with children from other major cities, including New York, Atlanta, Phoenix and Boston [8]. However, extrapolation of contact rates requires caution because the Bay Area differs from the broader USA in several dimensions: higher household income, higher educational attainment, larger workforce, smaller household sizes, smaller proportion of African Americans and higher compliance with social distancing [65]. During the spring semester, the Bay Area had a higher proportion of essential workers than the national average [45], which could translate into a larger impact of workplace closures in non-Bay Area cities. As we demonstrated, the impact of school closures varies by the proportion of households that have school-aged children, as well as the average school and class size of local public schools. Accordingly, the risk associated with school-based transmission will be higher in cities with a greater proportion of school-aged children, as well as larger school or classroom sizes. Nevertheless, many findings pertaining to school reopening are generalizable—such as teachers experiencing the greatest risks; high schools being at higher risk than elementary schools; high community transmission increasing risk in the absence of safety measures put in place; and the relative ranking of interventions. After all, key epidemiological parameters (e.g. susceptibility of children, asymptomatic transmission, mask effectiveness) apply across locations, and several population-level parameters (e.g. household size) apply to other urban areas.

Selection bias in our survey is possible because it was administered in English, and respondents were less likely to be essential workers. Discrepancies observed in the number of contacts by work location (outside versus inside the home) and ethnicity (Hispanic versus non-Hispanic) are thus expected to be biased towards the null. Our sample does not capture contact patterns among and between adults who do not have children, particularly missing those of young adults (18–29) or older adults (65+). However, our results are similar to estimates captured in another Bay Area contact survey that targeted households with and without children [8].

Community contacts under modelled school closure scenarios account for increases in daycare contacts only at the rates observed in our community survey, when fewer adults were permitted to work in-person. Therefore, modelled school closures or staggered weeks while reopening for a subsequent four-month semester may not adequately account for increases in community contacts from daycare settings. Similarly, the attributional effect of school reopening does not account for increases in workplace transmission that may occur if working parents return to in-person work once their child's school resumes in-person instruction

All of our modelled estimates depend, in part, on imperfectly understood epidemiological parameters, such as the relative susceptibility of children [21,64] and transmissibility of asymptomatic individuals [21,43,64]. We compare modelling results across various assumptions of each but contact tracing studies that seek to capture the relative susceptibility and infectiousness of symptomatically and asymptomatically infected children across ages are urgently needed.

While our model accounts for isolation of symptomatic individuals and quarantine of household members, modelled community interventions do not necessarily include the full effects of population-level contact tracing. However, based on modelled estimates of the effect of contact tracing used by the Bay Area over this period, we do not expect that our conclusions about school closures would change substantially if accounting for this [42,66]. While we found large reductions in risk with mask use and physical distancing, modelled within-school interventions did not include infection control measures, such as improved ventilation, increased handwashing, desk spacing or reduced sharing of supplies, which may further reduce transmission. Based on conversations about feasibility with school districts, we chose to model a periodic TTI intervention, in which testing was conducted on a monthly or weekly basis, rather than reactively based on symptom presentation. Other studies have demonstrated that reactive TTI can prevent a second transmission wave caused by school reopening [16].

## 5. Conclusion

Given the myriad individual and societal consequences of school closures, policymakers must urgently dedicate resources to support the package of interventions necessary to mitigate risk in schools. Focus should be placed first on reopening elementary schools, where a more limited set of interventions may be required, and risk of school-attributable transmission lower.

## Data accessibility

Data and code for this analysis can be found at: https://github.com/jrhead/COVIDandSchools.
